# Supplementary material for: Precipitation disaster hotspots depend on historical climate variability
Source: Nat Commun. 2025 Nov 29;17:16. doi: 10.1038/s41467-025-66601-2 (PMC12764945; doi:10.1038/s41467-025-66601-2)
Supplement: Supplementary file 1 — Supplementary Information [file 41467_2025_66601_MOESM1_ESM.pdf]

Supplementary Information to *Precipitation disaster hotspots depend on historical climate variability*

Iris de Vries* Maybritt Schillinger Erich Fischer Sebastian Sippel
Reto Knutti

*irisedevries@gmail.com

S1 Supplementary Note 1

Data information and checks
Model data

Model	Native resolution	# historical+SSP2-4.5 members
ACCESS-CM2 [1]	1.875° × 1.25°	5
ACCESS-ESM1-5 [2]	1.875° × 1.25°	40
CESM2 [3]	1.25° × 0.9375°	5
EC-Earth3 [4]	0.7° × 0.7°	19
EC-Earth3-Veg [5]	0.7° × 0.7°	8
HadGEM3-GC31-LL [6]	1.875° × 1.25°	5
MRI-ESM2-0 [7]	1.125° × 1.125°	5
UKESM1-0-LL [8]	1.875° × 1.25°	13

SI Table S1: CMIP6 models used in this study. The models are regridded to the resolutions of HadEX3 (1.875×1.25) and REGEN (1×1)

Autocorrelation

As mentioned in the main text, the temporal independence of Rx1d values is a prerequisite for temporal independence of record-breaking probabilities and validity of equations (1), (2) and (3). Fig. S1 shows that autocorrelations between Rx1d values at both high and low frequencies (short and long lags) are 0. The autocorrelation of the non-detrended values is positive and gradually decaying because there is a long term trend. This trend affects the record-breaking probabilities over time, but does not violate temporal independence of Rx1d values.
Dataset agreement

S2 Supplementary Note 2

Spatial pooling methods for observational/reanalysis GEV fits

As mentioned in Sect. 4.2, we tested two different spatial pooling methods to improve the GEV fits to the short observational and reanalysis timeseries. Below we outline the effects of shape-only and naive spatial pooling, focusing on the HadEX3-grid.

For shape-only spatial pooling, strength is borrowed from neighbouring gridcells to improve the estimate of ξ only [9]. In practice, we pool Rx1d data within a spatial window of 3×3 gridcells (5×5 for REGEN and ERA5) to fit a GEV, determining unique μ and σ values for each gridcell in the window, but allowing only one universal ξ value for all gridcells in the window. This is achieved by defining spatial covariates for μ and σ that, as it were, 'turn on' the individual gridcells in the fitting procedure,

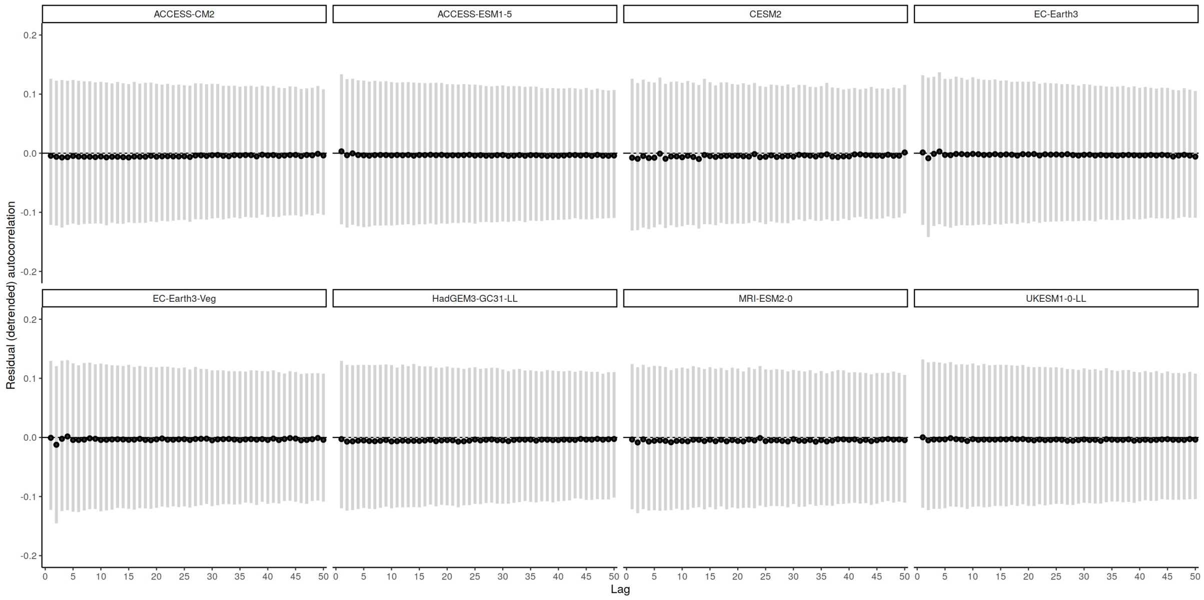

SI Figure S1: Temporal autocorrelation of detrended residuals of Rx1d for lags from 1 to 50, for all eight CMIP6 models used. Detrending was done by subtracting the loess-filtered ensemble mean timeseries per gridcell.

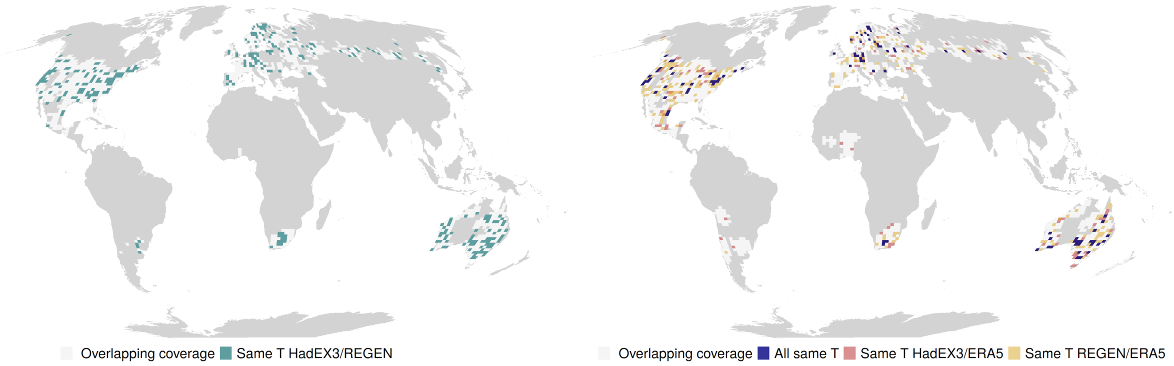

SI Figure S2: Gridcells where the different observational and reanalysis datasets show the same record-setting year T for the observational period 1950–2015

while ξ has no spatial covariate. The resulting GEV parameters are assigned to the middle gridcell of the window. Naive spatial pooling, on the other hand, implies we simply fit one GEV to all the Rx1d data pooled in a spatial window, and assign the GEV parameters to the middle gridcell, as done by e.g. Tradowsky et al. (2023) [10]. There is thus a degree of spatial smoothing for all three GEV parameters in this case, whereas the shape-only method aims at smoothing only ξ. Zeder et al (2024) [11] compares several GEV estimation methods including the two we test and finds that both are equally effective at improving accuracy of Rx1d return level estimates for return periods longer than the sample size. In our case, however, we aim to increase accuracy of return period estimates for events that did happen, and potentially have return periods shorter than the sample size.

Supplementary Fig. S3 shows the effect of the pooling schemes on the distribution of quantile levels in the observational and reanalysis datasets. The normalised density of record quantile levels using different GEV fitting methods is shown, and the grey shading shows the model mean envelope determined based on full-ensemble GEV distributions, which can be considered the highest-accuracy result. Both pooling methods shift the peak of the distribution to much higher quantile level values, and increase distribution sharpness. For observational/reanalysis datasets, the difference between naive and shape-only pooling is not very evident, but Supplementary Fig. S4 shows a systematic effect when we apply the different pooling schemes to climate models in the same way as we do to observations. Where naive pooling (green lines in Fig. S4) approximates the black full-ensemble curves quite closely, compared to single grid-cell,

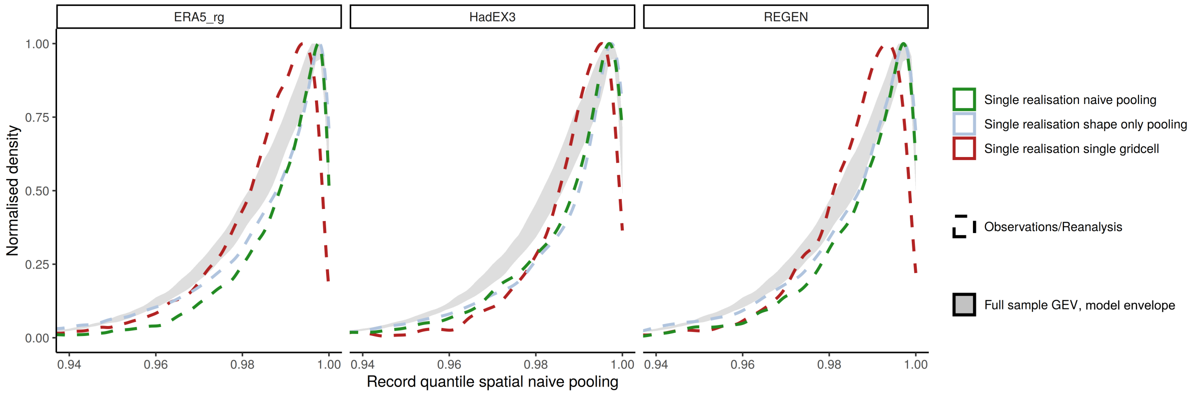

SI Figure S3: Normalised density plots of the historical record quantile levels determined using different GEV fitting methods. Dashed coloured lines correspond to different GEV fitting methods applied to the observational and reanalysis datasets (see subplot titles). In all plots, grey shading shows the envelope of 'true' record quantile levels determined from full-ensemble GEV fits to the climate models on the observational grid in question.

single realisation fits (red), shape-only pooling (blue) results in too large a correction to higher quantile levels and features too few moderate quantile levels between ≈0.98 and 0.995.

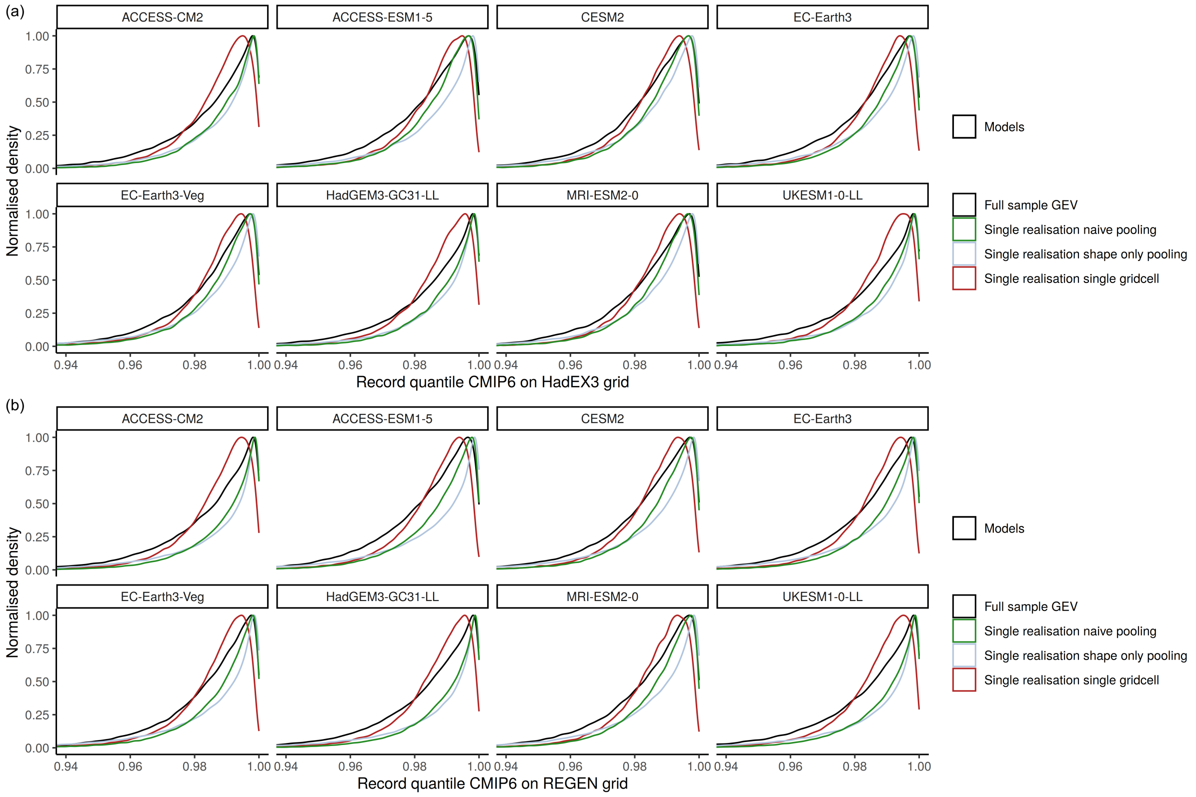

SI Figure S4: Normalised density plots of the historical record quantile levels determined using different GEV fitting methods to the different climate models, on the HadEX3 grid (a) and the REGEN and ERA5 grid (b). Coloured lines correspond to different 'as observations' GEV fitting methods, and the black line corresponds to the full-ensemble GEV fit of highest accuracy.

Shape-only pooling leads to smoother patterns and smaller magnitudes of ξ, which makes them more similar to the full-ensemble distribution's ξ, see Supplementary Fig. S5 column b. However, also σ responds strongly to this pooling scheme, since the setup aims to fit a σ and μ that are specific to single gridcell data. One might say that shape-only pooling partly decouples the fit of μ and σ from

the fit of ξ, where the μ and σ are fit with only part of the information (one gridcell). This leads to compensation effects in primarily the σ values, which strongly affect the estimated record quantile levels. In part of the gridcells, the compensation leads to decreases in σ relative to the single-gridcell fits, which are associated with strong increases in quantile levels estimated – the smaller the initial quantile, the stronger the increase. In another subset of gridcells, the compensation leads to increases in σ, which leads to decreasing quantile levels, especially for already lower ones. The combination of these two σ-related quantile level changes leads to clustering of most of the quantile levels at very high values, and some being moved to very low values. Therefore we see a certain "pulling apart" in the quantile level distribution which leaves a gap in the middle ranges where quantile levels between $\approx$0.98 and 0.995 should be found, see Supplementary Fig. S4. This effect is seen for the observational and reanalysis record quantile levels as well. Most of the quantile levels are overestimated in this approach, leading to an underestimation of record-breaking probabilities. We tried different shape-only pooling window sizes of 3×3 and 5×5 on the HadEX3 grid, and see that the 5×5 window size results in smoother shape parameters, more similar to those of the full-ensemble fit, however, the discrepancy in the quantile level distributions increases with increasing window size.

In the naive pooling setting, a coherent scale and shape parameter are fit to the pooled sample, leading to less local compensation effects of the scale parameter: we see much smaller changes relative to the single-gridcell fit, whereas the smoothing of the shape parameter is almost as effective as in the shape-only pooling setting. Supplementary Fig. S4 shows the most improvement in the density of record quantile levels for naive spatial pooling. Nonetheless, the frequency of moderate quantile levels remains too low compared to the full-ensemble fit for naive spatial pooling, explaining the biases in the CCP values shown in e.g. main Fig. 9e-g.

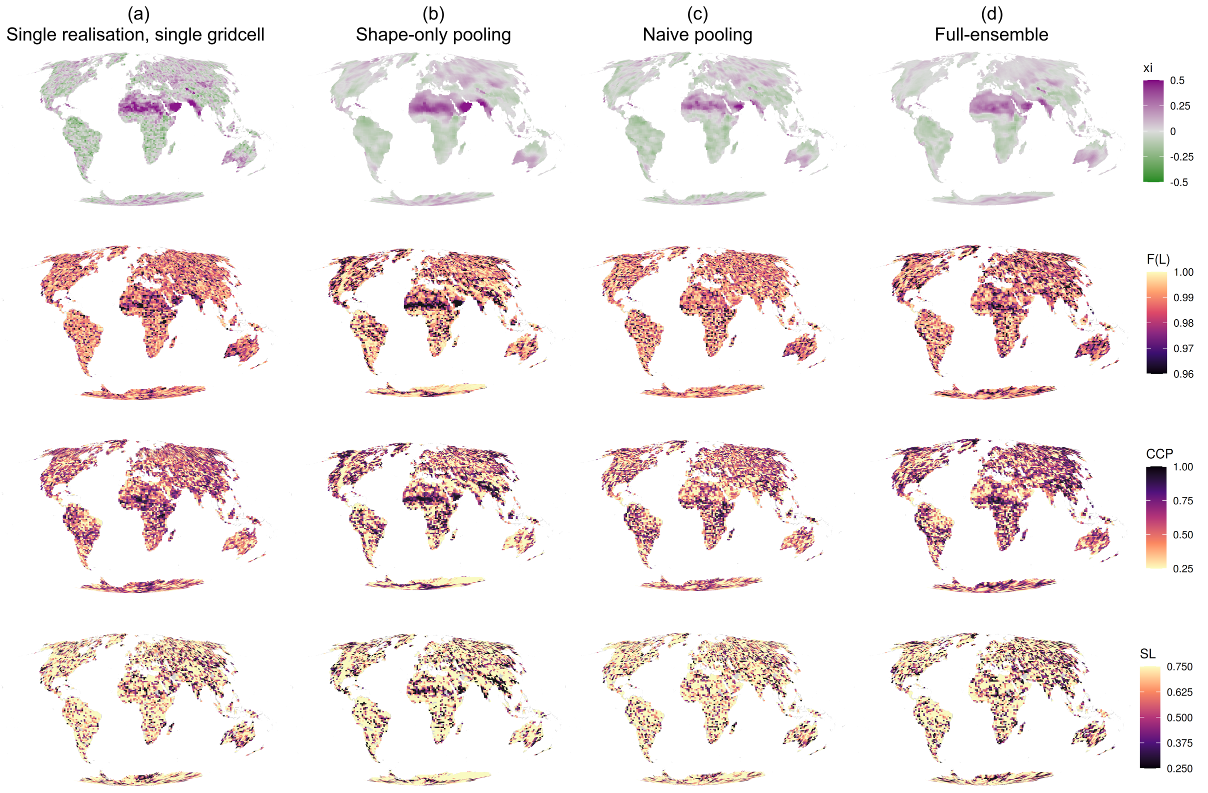

SI Figure S5: Patterns of GEV shape parameter ξ (a-d), historical record quantile level in the year of record occurrence (e-h), 2015 state likelihood (j-l), and PCC in 2050 (m-p). All patterns are from a single member randomly selected from the ACCESS-ESM1-5 ensemble. Shape parameter and historical record quantile levels are computed based on GEV fits following the method indicated above the columns. GEV for future quantile level evolution are in all cases based on the full ensemble, as in the main text, see 4.2 for details.

We perform a few additional tests to confirm the seemingly better performance of naive spatial pooling. As the aim of our study is to estimate record-breaking probabilities, we assess the skill of the probability estimate for each spatial pooling method using ranked probability skill scores (RPSS).

These are determined by estimating cumulative record-breaking probabilities treating model members as observations, and comparing the estimate to the actual future evolution of the model member; see Sect. 4.4 on validation for a full explanation. The RPSS represents the improvement of the estimation method in question relative to a benchmark. Table 1 in Sect. 4.4 shows RPSS values of the record-breaking probability estimates corresponding to different GEV-methods used to determine the record quantile levels. The first row refers to GEV distributions fitted to the 1950–2015 timeseries of single gridcells, where no optimisation has been done to reduce biases in the GEV fit due to the small sample size. We see a minor skill improvement of 5% over the benchmark. For shape-only spatial pooling, the improvement over the benchmark is in fact negative, i.e. the probability estimates are worse. GEV fits using naive spatial pooling lead to more than twice as much skill gain as the single gridcell GEV. We tested larger window sizes and see that a window size of 3×3 on the HadEX3 grid is better than larger windows. The probability estimates based on full ensemble GEV distributions are 30% more accurate than the benchmark.

Lastly, we confirm that not just bulk properties, but also the spatial pattern of ξ and the probability metrics improve most when naive spatial pooling is used. Supplementary Fig. S5 shows the ξ, record quantile level, state likelihood and 2050 CCP maps of one single member of ACCESS-ESM1-5 – the largest ensemble in our model selection. We show metrics based on single gridcell, shape-only pooled, naively pooled and full-ensemble GEV fits for visual comparison, and Supplementary table S2 provides the multi-model spatial correlations of these quantities obtained from the different 'as observations' GEV fits to those obtained using the full-ensemble GEV.

The maps and spatial correlations show a much stronger agreement of the naive pooling GEV based results with the full-ensemble GEV based results. For shape-only pooling, strong patterning appears that seems influenced by the climatology and leads to artificial regions of low state likelihood and high future record-breaking probability.

Given the clearly better performance (for our purposes) of naive spatial pooling in all tests performed, we employ naive spatial pooling for the observational/reanalysis GEV fits in our analysis.

SI Table S2: Spatial correlation coefficients of the variables listed in the top row; correlations of the result obtained using the GEV fitting method listed in the first column with the result obtained using the full-ensemble GEV. SL refers to state likelihood: $1 - \Pr(T < Y_{\mathrm{NR}} \leq 2015|L)$ and CCP to $\Pr(Y_{\mathrm{NR}} \leq 2050|L, T = 2015)$

**GEV fitting method**	ξ	μ	σ	$F(L)$	SL	CCP
Single gridcell 'as obs' GEV	0.70	0.89	0.97	0.43	0.69	0.57
Shape-only spatial pooling GEV, 3×3 window	0.79	0.85	0.91	0.53	0.68	0.59
Naive spatial pooling GEV, 3×3 window	0.75	0.93	0.98	0.75	0.85	0.79

S3 Supplementary Note 3

Supplementary figure to main Fig. 3d-e

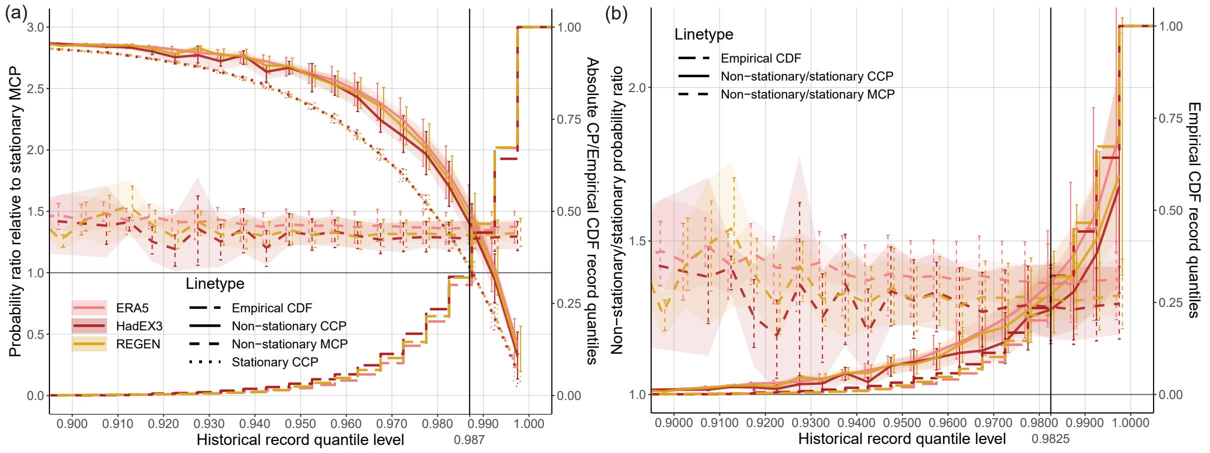

SI Figure S6: As main Fig. 3d-e, but with empirical (non-parametric) CDF per quantile level bin (on right y-axis), showing fraction of gridcells subject to the corresponding probability ratios. Error bars show interquartile range of all gridcells in bin in question.

S4 Supplementary Note 4

Derivation quantile level of records

Intuitively, the expected quantile level of the maximum M_j of an i.i.d. sample $\{X_1, \ldots, X_j\}$ of length j should correspond to $1 - \frac{1}{j+1}$. This follows from the fact that the marginal record-breaking probability at timestep j is $\frac{1}{j}$. For example, for timestep $j = 3$, the average record-breaking rate is $\frac{1}{3}$, meaning that the quantile level of the current record at time $j = 2$ is $1 - \frac{1}{3}$. This means that the record set at time $j = 1$ has an average quantile level of 0.5, hence, $1 - \frac{1}{j+1}$.

Formally, we derive this result as follows. We are looking for the quantile level of the maximum M_j, i.e. $F_X(M_j)$, where F_X is the CDF of each of the i.i.d. data points X_i and M_j is the maximum of $X_1, \ldots, X_j$. In order to find the expected value of $F_X(M_j)$, we need an expression for the PDF of $F_X(M_j)$, which we find by determining the CDF of $F_X(M_j)$ in equation (1) and taking the derivative of that expression in equation (2).

$$
\begin{aligned}
&F(F_X(M_j)) = \Pr(F_X(M_j) \leq F_X(m)) = \\
&\qquad \Pr(M_j \leq m) = \Pr(\max(X_1, \ldots, X_j) \leq m) = \Pr(X_1 \leq m) \cdot \cdots \cdot \Pr(X_j \leq m) = F_X(m)^j \qquad (1)
\end{aligned}
$$

In the equation above we use the property that $\Pr(F_X(M_j) \leq F_X(m)) = \Pr(M_j \leq m)$. To find the PDF for $F_X(M_j)$, we take the derivative of the previous expression:

$$f(F_X(M_j)) = jF_X(m)^{j-1} f_X(m) \qquad (2)$$

Now we can determine the expected value of $F_X(M_j)$ as follows:

$$\mathbb{E}[F_X(M_j)] = \int F_X(m) f(F_X(M_j)) dm = \int F_X(m) jF_X(m)^{j-1} f_X(m) dm = j \int F_X(x)^j f_X(x) dx \qquad (3)$$

We can substitute $u = F_X(x)$ in the previous integral and instead of integrating $f_X(x)dx$ we then integrate over du from 0 to 1, leading to the final result:

$$\mathbb{E}[F_X(M_j)] = j \int_0^1 u^j du = j \cdot \left[\frac{1}{j+1} u^{j+1}\right]_0^1 = \frac{j}{j+1} = 1 - \frac{1}{j+1} \qquad (4)$$

Note: the above holds if $\{X_1, ..., X_j\}$ is a stationary, i.i.d. sample. This property manifests in the stationary CCP/MCP ratios in Fig. 3d-e.

S5 Supplementary Note 5

Marginal cumulative record-breaking probability patterns

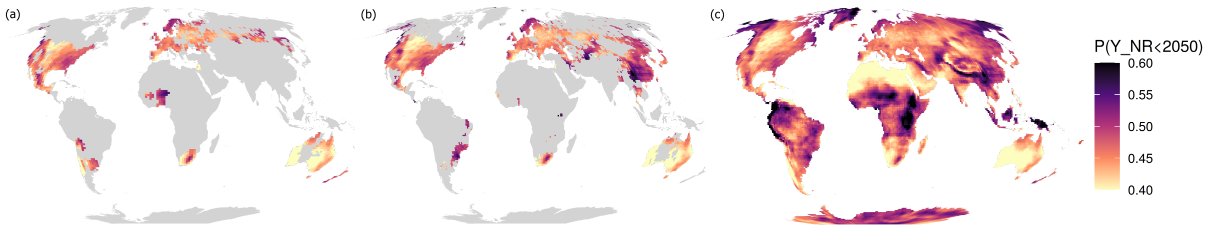

SI Figure S7: Marginal cumulative record-breaking probability (MCP) by 2050 as defined in the main text for HadEX3 (a), REGEN (b) and ERA5 (c).

Figure S7 shows the MCP by 2050 for the observational and reanalysis datasets. The MCP includes only the effect of climate change on record-breaking probabilities, and is independent of the historical record level to be exceeded. The difference in magnitude between HadEX3 and REGEN on the one hand and ERA5 on the other hand is due to the start year; for HadEX3 and REGEN the cumulative summation starts in 2016, for ERA5 in 2024.

S6 Supplementary Note 6

Additional validation state likelihood

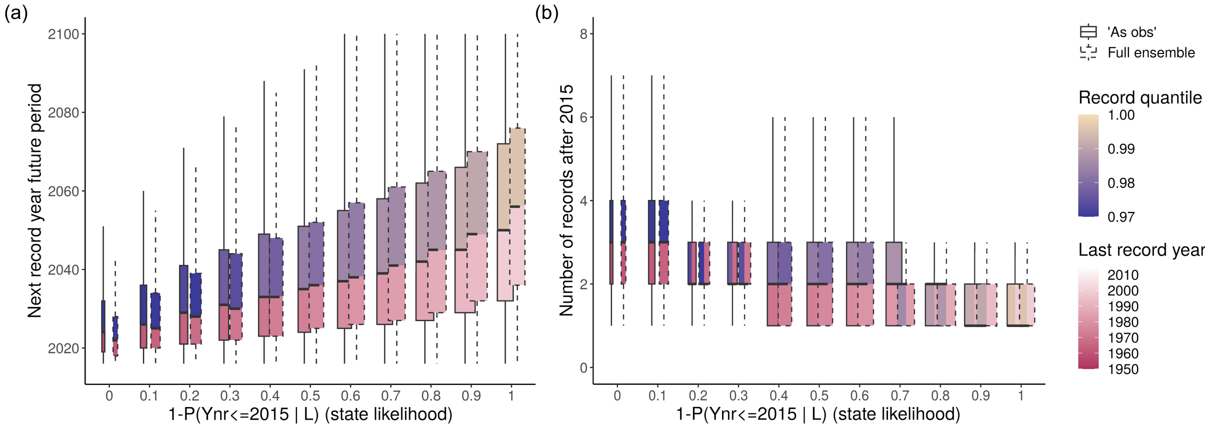

SI Figure S8: Correlation of the state likelihood (binned) in 2015 with the year of next record occurrence (a) and with the number of records in the period 2016–2100 (B), evaluated in all CMIP6 models. The colour shading of the bars show the associated bin-means of record quantile level and record-setting year T, with a clear gradient towards lower record quantile levels (less extreme) and longer-ago years as the state likelihood decreases.

Fig. S8 shows the correlation between state likelihood and indicators of future record-breaking in CMIP6 models, in part validating its use as an indicator of disaster potential.

Fig. S9 shows that the selected single station data in the regions discussed in the main text shows a clear signature corresponding to the events we associated with the record-breaking. These stations were selected based on their location being in the gridcells of interest; gridcells with 2009 state likelihood ≤ 0.25 and record-breaking in the years 2010–2015, and reported in the disaster reports of the events in question [12–15]. Not all stations show the maximum daily event, which is expected as HadEX3 aggregates multiple stations in their Rx1d product, but all stations show daily precipitation values in the uppermost quantile levels of the full sample. Storm Agatha (San Salvador) is least well represented, also in other stations in the region that we assessed. The lower data quality in Central America plays a big part (exemplified by the sparsity of the record for San Salvador): this impairs confidence in both the accuracy of records in HadEX3 as well in the verification data itself.

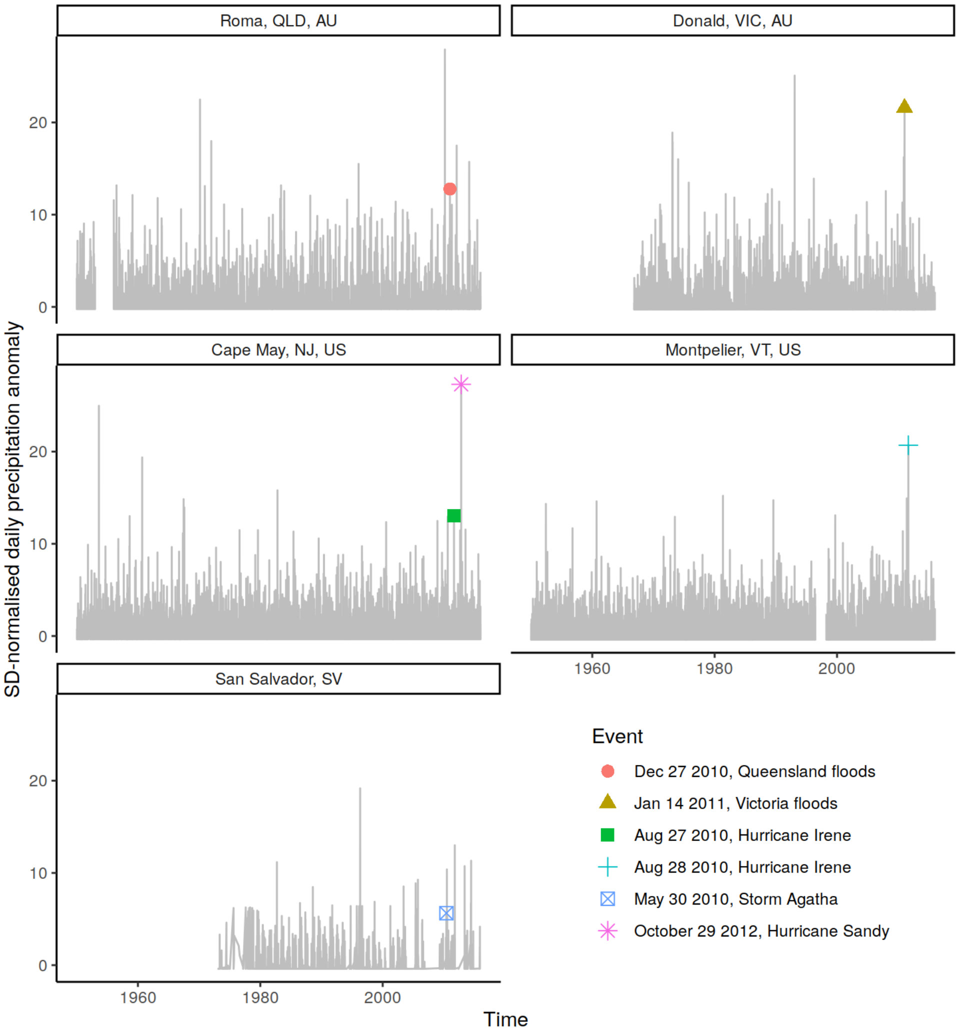

SI Figure S9: Daily precipitation observations from single stations from the GHCN-Daily network [16–19] and the Australian Bureau of Meteorology [20, 21]. Events mentioned in the main text are indicated with markers [12–15].

For reference, we added a marker for Hurricane Sandy in the timeseries for Cape May, which evidently led to record-breaking precipitation in New Jersey, where it made landfall. This event of October 2012 is not clearly visible as record-breaking cluster in HadEX3, which we hypothesise could be associated with the single landfall of Sandy, as opposed to the repeated 'bouncing' landfall of Irene.

Supplementary References

[1] Dix, M., Bi, D., Dobrohotoff, P., Fiedler, R., Harman, I., Law, R., Mackallah, C., Marsland, S., O'Farrell, S., Rashid, H., Srbinovsky, J., Sullivan, A., Trenham, C., Vohralik, P., Watterson, I., Williams, G., Woodhouse, M., Bodman, R., Dias, F.B., Domingues, C.M., Hannah, N., Heerdegen, A., Savita, A., Wales, S., Allen, C., Druken, K., Evans, B., Richards, C., Ridzwan, S.M., Roberts, D., Smillie, J., Snow, K., Ward, M., Yang, R.: CSIRO-ARCCSS ACCESS-CM2 model output prepared for CMIP6 ScenarioMIP. Earth System Grid Federation (2019). https://doi.org/10.22033/ESGF/CMIP6.2285 . https://doi.org/10.22033/ESGF/CMIP6.2285

[2] Ziehn, T., Chamberlain, M., Lenton, A., Law, R., Bodman, R., Dix, M., Wang, Y., Dobrohotoff, P., Srbinovsky, J., Stevens, L., Vohralik, P., Mackallah, C., Sullivan, A., O'Farrell, S., Druken, K.: CSIRO ACCESS-ESM1.5 model output prepared for CMIP6 ScenarioMIP. Earth System Grid Federation (2019). https://doi.org/10.22033/ESGF/CMIP6.2291 . https://doi.org/10.22033/ESGF/CMIP6.2291

[3] Danabasoglu, G.: NCAR CESM2 model output prepared for CMIP6 ScenarioMIP. Earth System Grid Federation (2019). https://doi.org/10.22033/ESGF/CMIP6.2201 . https://doi.org/10.22033/ESGF/CMIP6.2201

[4] EC-Earth Consortium (EC-Earth): EC-Earth-Consortium EC-Earth3 model output prepared for CMIP6 ScenarioMIP. Earth System Grid Federation (2019). https://doi.org/10.22033/ESGF/CMIP6.251 . https://doi.org/10.22033/ESGF/CMIP6.251

[5] EC-Earth Consortium (EC-Earth): EC-Earth-Consortium EC-Earth3-Veg model output prepared for CMIP6 ScenarioMIP. Earth System Grid Federation (2019). https://doi.org/10.22033/ESGF/CMIP6.727 . https://doi.org/10.22033/ESGF/CMIP6.727

[6] Good, P.: MOHC HadGEM3-GC31-LL model output prepared for CMIP6 ScenarioMIP. Earth System Grid Federation (2019). https://doi.org/10.22033/ESGF/CMIP6.10845 . https://doi.org/10.22033/ESGF/CMIP6.10845

[7] Yukimoto, S., Koshiro, T., Kawai, H., Oshima, N., Yoshida, K., Urakawa, S., Tsujino, H., Deushi, M., Tanaka, T., Hosaka, M., Yoshimura, H., Shindo, E., Mizuta, R., Ishii, M., Obata, A., Adachi, Y.: MRI MRI-ESM2.0 model output prepared for CMIP6 ScenarioMIP. Earth System Grid Federation (2019). https://doi.org/10.22033/ESGF/CMIP6.638 . https://doi.org/10.22033/ESGF/CMIP6.638

[8] Good, P., Sellar, A., Tang, Y., Rumbold, S., Ellis, R., Kelley, D., Kuhlbrodt, T., Walton, J.: MOHC UKESM1.0-LL model output prepared for CMIP6 ScenarioMIP. Earth System Grid Federation (2019). https://doi.org/10.22033/ESGF/CMIP6.1567 . https://doi.org/10.22033/ESGF/CMIP6.1567

[9] Cooley, D., Hunter, B.D., Smith, R.L.: Univariate and multivariate extremes for the environmental sciences. In: Handbook of Environmental and Ecological Statistics, pp. 153–180. Chapman and Hall/CRC, New York (2019). https://doi.org/10.1201/9781315152509-9

[10] Tradowsky, J.S., Philip, S.Y., Kreienkamp, F., Kew, S.F., Lorenz, P., Arrighi, J., Bettmann, T., Caluwaerts, S., Chan, S.C., De Cruz, L., de Vries, H., Demuth, N., Ferrone, A., Fischer, E.M., Fowler, H.J., Goergen, K., Heinrich, D., Henrichs, Y., Kaspar, F., Lenderink, G., Nilson, E., Otto, F.E.L., Ragone, F., Seneviratne, S.I., Singh, R.K., Skålevåg, A., Termonia, P., Thalheimer, L., van Aalst, M., Van den Bergh, J., Van de Vyver, H., Vannitsem, S., van Oldenborgh, G.J., Van Schaeybroeck, B., Vautard, R., Vonk, D., Wanders, N.: Attribution of the heavy rainfall events leading to severe flooding in Western Europe during July 2021. Climatic Change **176**(7), 90 (2023) https://doi.org/10.1007/s10584-023-03502-7

[11] Zeder, J., Fischer, E.M.: Decadal to centennial extreme precipitation disaster gaps — long-term variability and implications for extreme value modelling. Weather and Climate Extremes **43**, 100636 (2024) https://doi.org/10.1016/j.wace.2023.100636

[12] Queensland Floods Commission of Inquiry: Final report. https://knowledge.aidr.org.au/media/3456/qfci-final-report-march-2012.pdf (2012)

[13] Comrie, N.: Review of the 2010-11 flood warnings and response - Final report. https://knowledge.aidr.org.au/media/4456/review-of-the-2010-11-flood-warnings-plus-response_victoria.pdf (2011)

[14] Avila, L.A., Cangialosi, J., National Hurricane Center: Tropical Cyclone Report Hurricane Irene. https://www.nhc.noaa.gov/data/tcr/AL092011_Irene.pdf. Accessed 13 May 2024 (2011)

[15] Beven, J.L., National Hurricane Center: Tropical Cyclone Report Tropical Storm Agatha. https://www.nhc.noaa.gov/data/tcr/EP012010_Agatha.pdf. Accessed 09 May 2024 (2010)

[16] Menne, M.J., Durre, I., Korzeniewski, B., McNeill, S., Thomas, K., Yin, X., Anthony, S., Ray, R., Vose, R.S., E.Gleason, B., , Houston, T.G.: Global Historical Climatology Network - Daily (GHCN-Daily), Version 3, station Cape May NAS, NJ, USA. Accessed 18 November 2024 (2012). https://doi.org/10.7289/V5D21VHZ

[17] Menne, M.J., Durre, I., Korzeniewski, B., McNeill, S., Thomas, K., Yin, X., Anthony, S., Ray, R., Vose, R.S., E.Gleason, B., , Houston, T.G.: Global Historical Climatology Network - Daily (GHCN-Daily), Version 3, station Edward F Knapp State Airport, VT, USA. Accessed 18 November 2024 (2012). https://doi.org/10.7289/V5D21VHZ

[18] Menne, M.J., Durre, I., Korzeniewski, B., McNeill, S., Thomas, K., Yin, X., Anthony, S., Ray, R., Vose, R.S., E.Gleason, B., , Houston, T.G.: Global Historical Climatology Network - Daily (GHCN-Daily), Version 3, station San Salvador/Ilopan, El Salvador. Accessed 18 November 2024 (2012). https://doi.org/10.7289/V5D21VHZ

[19] Menne, M.J., Durre, I., Vose, R.S., Gleason, B.E., Houston, T.G.: An overview of the global historical climatology network-daily database. J. Atmos. Ocean. Technol. **29**(7), 897–910 (2012)

[20] Australian Government Bureau of Meteorology: Daily Rainfall Climate Data, station number 43093, Waverley Downs, Queensland. http://www.bom.gov.au/climate/data/stations/. Accessed 18 November 2024

[21] Australian Government Bureau of Meteorology: Daily Rainfall Climate Data, station number 78072, Donald, Victoria. http://www.bom.gov.au/climate/data/stations/. Accessed 18 November 2024
